# Mortality and prognostic factors for spontaneous pneumothorax in older adults

**DOI:** 10.1371/journal.pone.0291233

**Published:** 2023-09-08

**Authors:** Saori Nishizawa, Kazunori Tobino, Yousuke Murakami, Kazuki Uchida, Takafumi Kawabata, Hiroyuki Ota, Yuri Hiramatsu, Takuto Sueyasu, Kosuke Tsuruno

**Affiliations:** Department of Respiratory Medicine, Iizuka Hospital, Iizuka, Fukuoka, Japan; Gulu University, UGANDA

## Abstract

Spontaneous pneumothorax occurs predominantly in young males and older adults, often as a secondary condition, and can be refractory and fatal. This study aimed to investigate the mortality and prognostic factors for pneumothorax in older patients. We retrospectively cohort studied patients with pneumothorax aged ≥65 years who visited our department from October 2012 to January 2019. Data on sex, age, medical history, smoking history, underlying lung disease, treatment, and prognosis were extracted from medical records. Cox proportional hazards regression analysis was used to investigate pneumothorax mortality and prognostic factors. In total, 239 patients were included. Among them, 36 (15%) died during hospitalization. Respiratory disease was the direct cause of death in 30 patients (83.3%), and 211 (88.3%) patients had underlying lung disease. The incidence of pneumonia in our hospital was 22.6% (54 cases). On admission, the mortality rate was 33% (18/54) in patients with concomitant pneumonia; univariate analysis showed significant differences in the Charlson Comorbidity Index (CCI), activities of daily living (ADL), and concomitant pneumonia. In the Cox proportional hazards analysis of ADL (p = 0.09), CCI (p = 0.05), and concomitant pneumonia on admission (p = 0.02), concomitant pneumonia on admission was found to be an independent predictor of in-hospital mortality. This study suggests that concomitant pneumonia at admission may be a mortality risk factor for pneumothorax.

## Introduction

Spontaneous pneumothorax, also known as primary spontaneous pneumothorax (PSP), most commonly occurs in young people with no underlying pulmonary disease. It is secondary to chronic obstructive lung disease (COPD), cystic fibrosis, pneumocystis pneumonia, lung cancer, and interstitial lung diseases (ILDs) in older adults. In a study of pneumothorax in older adults in Japan, 90% had underlying lung disease [1], referred to as secondary spontaneous pneumothorax (SSP) [2, 3]. SSP is a potentially life-threatening disease because of impaired lung function or general condition, due to underlying lung disease. The presence of underlying lung disease often makes pneumothorax more refractory and difficult to treat, thus prolonging hospitalization and decreasing activities of daily life (ADL). Onuki et al. reported that the mortality rate of 751 patients with spontaneous pneumothorax was 1.7%, of which 4.6% (266) were patients with SSP [4]. They reported that emphysema was the most frequent cause of death in SSP patients. However, to the best of our knowledge, there are no reports in Japan on the prognostic factors of spontaneous pneumothorax in the older population. Japan’s super-aging society and the need to consider palliative management based on the patient’s underlying disease and general condition necessitate this study. Therefore, this study aimed to investigate the mortality and prognostic factors of spontaneous pneumothorax in older adults.

## Materials and methods

### Patients and research ethics

Consecutive patients aged ≥65 years with spontaneous pneumothorax who visited our department from October 1st, 2012 to January 31st, 2019 were retrospectively studied. This study was performed from September 1, 2021, to March 31, 2022. We had access to information that could identify individual participants during or after data collection. This Clinical Research study was approved by the Institutional Review Board of Iizuka Hospital (approval no. 21085) and the ethics committee waived the requirement for informed consent. Participants were provided with detailed information about the study objectives, procedures, and potential risks and benefits, which was available on the study website. All study procedures were conducted in accordance with the ethical standards of the institutional and/or national research committee and with the 1964 Helsinki Declaration and its later amendments or comparable ethical standards.

### Data and statistical analyses

Data extracted from the medical records included age, sex, smoking status, ADL before admission (complete independence, total assistance, and others), history of pneumothorax, regular use of systemic corticosteroids, use of home oxygen therapy, time from the onset of pneumothorax to a hospital visit, comorbidities, length of hospitalization, management of pneumothorax, length of chest tube placement, intensive care unit admission, and survival. Since many older patients had not been diagnosed with COPD at the time of admission, we categorized patients based on the presence or absence of emphysema on CT scans rather than using COPD as the underlying pulmonary disease. In addition, chest radiographs on admission were reviewed, and the size of the pneumo-thorax was classified according to the Japan Society for Pneumothorax and Cystic Lung Diseases criteria [5], the American College of Chest Physicians [6], and the British Thoracic Society [7]. Management of pneumothorax included observation, needle aspiration, chest tube drainage, chemical pleurodesis, surgery, thoracographic fibrin glue sealing method, and endobronchial Watanabe spigot placement, with some patients receiving multiple treatments.

We conducted a retrospective cohort study based on the medical records. All statistical analyses were performed using the EZR [8], a modified version of R commander designed to add statistical functions frequently used in biostatistics. The baseline characteristics were analyzed using descriptive statistics. Mann–Whitney’s U and Fisher’s exact tests were used to compare patient characteristics and treatment outcomes. Categorical variables were described as frequencies and percentages. Continuous variables with standard distributions were represented as median and standard derivations. We divided the participants into in-hospital death and survival groups. Univariate analysis was performed on the data extracted from the medical records. Cox proportional hazards regression analysis was performed on the univariate analyses that were significantly different; furthermore, we developed a Kaplan‒Meier survival model.

## Results

### Patient characteristics

Table 1 shows the patients’ clinical characteristics and a comparison of the survival and in-hospital mortality groups. A total of 239 patients (203 males and 36 females) with a median age of 79 years were included in the analysis. Of the 239 patients, 225 (94.1%) were hospitalized, and 36 (15.1%) died during hospitalization. Forty-six patients (19.2%) had a history of at least one pneumothorax event. There was a trend towards a slightly older age in the in-hospital death group compared with that in the survivor group (median age, 81 vs. 79, p = 0.08). No sex differences were observed between the two groups (p = 0.62). There was no statistically significant difference in smoking history and smoking status between the survival and in-hospital death groups (p = 0.89 for both). However, there was a statistically significant difference in ADL between the two groups, with more patients requiring some form of assistance in the in-hospital death group (p = 0.02). The number of patients with a history of pneumothorax was higher in the survival group (p < 0.01), and there was no significant difference between the two groups in the rate of regular systemic steroid use (p >0.05). The use of home oxygen therapy tended to be slightly higher in the in-hospital death group (p = 0.08), and the Charlson Comorbidity Index (CCI) was significantly higher in the in-hospital death group (p < 0.01). In addition, no statistically significant difference was observed between the two groups in terms of the time from the onset of pneumothorax to the hospital visit (p = 0.10), the number of hospitalized patients (p = 0.14), and the length of hospitalization (p = 0.75). The length of chest tube placement and intensive care unit admission rates were significantly longer and higher in the in-hospital death group (p = 0.02 and p = 0.03, respectively).

**Table 1:** Patient characteristics (N = 239).

Characteristics		Total (N = 239)	In-hospital death		*P*-value
			- (N = 203)	+ (N = 36)	
Age, Years		79 (72–85)	79 (71–84)	81 (76–87)	0.08
Sex	Male	203 (84.9)	171 (84.2)	32 (88.9)	0.62	
	Female	36 (15.6)	32 (15.8)	4 (11.1)		
Smoking history, pack-year		40 (2–60)	20 (13–60)	35 (5–63)	0.89
Smoking status	Current smoking	28 (11.7)	25 (12.4)	3 (8.3)	0.89
	Formerly smoked	148 (61.9)	125 (62.2)	23 (63.9)	
	Never smoked	54 (22.6)	46 (22.9)	8 (22.2)	
	Unknown	9 (3.8)	7 (3.5)	2 (5.6)	
Activities of daily living	Complete Independence	158 (66.1)	141 (69.5)	17(47.2)	0.02
	Total assistance	40 (16.7)	29 (14.3)	11 (30.6)	
	Other	41 (17.2)	33 (16.3)	8 (22.2)	
History of pneumothorax		46 (19.2)	45 (22.2)	1 (2.8)	< 0.01
Systemic corticosteroid use		31 (13.0)	27 (13.1)	4 (11.1)	1
Home oxygen therapy		26 (10.9)	19 (9.4)	7 (19.4)	0.08
Time from onset of pneumothorax to hospital visit, days		0 (0)	0 (0–1)	0 (0)	0.10
Charlson Comorbidity Index		2 (1–4)	2 (1–3)	3 (2–5)	<0.01
Hospitalised patients		225 (94.1)	189 (93.1)	36 (100)	0.14
Length of hospitalisation, days		21 (11–33)	21 (11–31)	22 (9–42)	0.75
Length of chest tube placement, days		10 (1–11)	6 (1–10.5)	9 (2.75–17.5)	0.02
ICU admission		2 (0.8)	0 (0)	2 (5.6)	0.03
CAUSES OF DEATH					
Pneumonia				16 (44.4)	
ILD				6 (16.7)	
COPD				3 (8.3)	
Pneumothorax				2 (5.5)	
Lung cancer				2 (5.5)	
Heart failure				2 (5.5)	
Other				5 (13.9)	

Age, smoking history, time from onset of pneumothorax to the hospital visit, Charlson Comorbidity Index, and length of hospitalization are presented as median (interquartile range). All the other data are presented as the number of patients (percentage of all patients).

COPD: chronic obstructive pulmonary disease, ILD: interstitial lung disease.

### Cause of death

Thirty-six patients (15.1%) died during hospitalization. Table 1 shows the cause of death determined for the 36 patients according to the direct cause recorded on the death certificate and summary. The causes of death were as follows: pneumonia, 16 patients (44.4%); ILD, six patients (16.7%); COPD, three patients (8.3%); pneumothorax, three patients (8.3%); lung cancer, two patients (5.5%); heart failure, two patients (5.5%); and other, five patients (13.9%). Respiratory disease was the cause of death in 30 patients (83.3%). Patients were admitted with pneumothoraxes, but many died because of deterioration of the underlying lung disease.

### Underlying lung diseases

Table 2 summarizes the underlying lung diseases. We opted to consider emphysema instead of COPD in this study due to the fact that many older patients are not diagnosed with COPD upon hospital admission. Only 28 (11.7%) patients had no underlying lung disease. A wide variety of underlying lung diseases were observed (emphysema, ILD, lung cancer, aspergillosis, asthma, tuberculosis, nontuberculous mycobacteriosis, pneumoconiosis, bronchiectasis, metastatic lung tumor, and Birt–Hogg–Dubé syndrome), but the frequency of these diseases did not differ significantly between the survival and in-hospital death groups. However, the frequency of comorbid pneumonia on admission was higher in the death group than that in the survival group (50.0% vs. 17.7%, p < 0.01).

**Table 2:** Underlying lung diseases.

Underlying lung diseases			Total N = 239	In-hospital death		*P*-value
				- (N = 203)	+ (N = 36)	
None			28 (11.7)	24 (11.8)	4 (11.1)	1
Emphysema	Emphysema only		94 (39.3)	82 (40.4)	12 (33.3)	0.46
	+ ILD	only	21 (8.8)	17 (8.4)	4 (11.1)	0.53
		+ Lung cancer	2 (0.8)	1(0.5)	1(2.8)	0.28
		+ Aspergillosis	1 (0.4)	1 (0.5)	0 (0)	1
		+ Asthma	1 (0.4)	1 (0.5)	0 (0)	1
		+ Chronic Tuberculosis	1 (0.4)	1 (0.5)	0 (0)	1
	+ Lung cancer	12 (5.0)	9 (4.4)	3 (8.3)	0.39
	+ Asthma	8 (3.3)	8 (3.9)	0 (0)	0.61
	+ Chronic tuberculosis	8 (3.3)	8 (3.9)	0 (0)	0.61
	+ Pneumoconiosis	only	5 (2.1)	4 (2.0)	1 (2.8)	0.56
		+ Asthma	1 (0.4)	1 (0.5)	0 (0)	1
	+ Bronchiectasis	2 (0.8)	2 (1.0)	0 (0)	1
	+ Aspergillosis	4 (1.7)	2 (1.0)	2 (5.6)	0.11
	+ Metastatic lung tumor	3 (1.3)	2 (1.0)	1 (2.8)	0.39
	+ BHD syndrome	2 (0.8)	2 (1.0)	0 (0)	1
ILD	ILD only		20 (8.4)	15 (7.4)	5 (13.9)	0.20
	+ Asthma		1 (0.4)	1 (0.5)	0 (0)	1
	+ Bronchiectasis	only	4 (1.7)	4 (2.0)	0 (0)	1
		+ NTM	1 (0.4)	0 (0)	1 (2.8)	0.15
Lung cancer			2 (0.8)	1 (0.5)	1 (2.8)	0.28
Asthma			5 (2.1)	5 (2.5)	0 (0)	1
Bronchiectasis	Bronchiectasis only		3 (1.3)	3 (1.5)	0 (0)	1
	+ NTM		3 (1.3)	3 (1.5)	0 (0)	1
	+ Pneumoconiosis		1 (0.4)	1 (0.5)	0 (0)	1
Chronic tuberculosis	Chronic tuberculosis only		2 (0.8)	1 (0.5)	1 (2.8)	0.28
	+ Aspergillosis		1 (0.4)	1 (0.5)	0 (0)	1
Pneumoconiosis			3 (1.3)	3 (1.5)	0 (0)	1
Concomitant pneumonia on admission			54 (22.6)	36 (17.7)	18 (50.0)	< 0.01

Data are presented as the number of patients (percentage of all patients).

BHD syndrome: Birt–Hogg–Dubé syndrome, ILD: interstitial lung disease, NTM: nontuberculous mycobacteriosis.

### Radiographic characteristics of pneumothoraxes

Table 3 shows the radiographic characteristics of pneumothoraxes. There was almost no difference between the disease on the left and right sides, and no significant association with in-hospital mortality. Regarding the size of pneumothorax, there was a poor association between the degree of collapse and in-hospital mortality for any criterion.

**Table 3:** Radiographic characteristics of pneumothorax.

Characteristics		Total N = 239	In-hospital death		*P*-value
			- (N = 203)	+ (N = 36)	
Side of pneumothorax	Left	104 (43.5)	90 (44.3)	14 (38.9)	0.59
	Right	135 (56.5)	113 (55.7)	22 (61.1)	
Size of pneumothorax					
ACCP	Small	167 (69.9)	139 (68.5)	28 (77.8)	0.33
	Large	72 (30.1)	64 (31.5)	8 (22.2)	
BTS	Small	135 (56.5)	114 (56.2)	21 (58.3)	0.86
	Large	104 (43.5)	89 (43.8)	15 (41.7)	
The Japan Society for Pneumothorax and Cystic Lung Diseases	Mild	59 (24.7)	50 (24.6)	9 (25.0)	0.16
	Moderate	126 (52.7)	103 (50.7)	23 (63.9)	
	Severe	54 (22.6)	50(24.6)	4 (11.1)	

Data are presented as the number of patients (percentage of all patients).

### Management of spontaneous pneumothorax

Table 4 shows the details of these treatments. Approximately 75% and 80% of patients with pneumothorax and those in the in-hospital death group respectively, received some treatment. For the management of pneumothorax, there was no significant difference between the two groups, except for zero surgical cases in the in-hospital death group. However, a treatment combining multiple methods was used more often in the survival group (15 patients vs. one patient).

**Table 4:** Management of pneumothorax.

Characteristics	Total N = 239	In-hospital death		*P*-value
		- (N = 203)	+ (N = 36)	
(a)Observation	58 (24.7)	51 (25.1)	7 (19.4)	0.53
(b)Needle aspiration only	6 (2.5)	5 (2.5)	1 (2.8)	1
(c)Chest tube drainage only	111 (46.4)	89 (43.8)	22 (61.1)	0.07
(d)Chemical pleurodesis	20 (8.4)	15 (7.4)	5 (13.9)	0.20
(e)Thoracographic fibrin glue sealing method only	1 (0.4)	1 (0.5)	0 (0)	1
(f)Endobronchial Watanabe spigot placement only	1 (0.4)	1 (0.5)	0 (0)	1
(g)Surgery	26(10.9)	26 (12.8)	0 (0)	0.02
(d) + (e)	5 (2.1)	4 (2.0)	1 (2.8)	0.56
(d) + (f)	1 (0.4)	1 (0.5)	0 (0)	1
(d) + (g)	3(1.3)	3 (1.5)	0 (0)	1
(d) + (e) + (f)	4 (1.7)	4 (2.0)	0 (0)	1
(d) + (e) + (g)	0 (0)	0 (0)	0 (0)	-
(d) + (e) + (f) + (g)	1 (0.4)	1 (0.5)	0 (0)	1
(d) + (f) + (g)	0 (0)	0 (0)	0 (0)	-
(e) + (f)	0 (0)	0 (0)	0 (0)	-
(e) + (g)	1 (0.4)	1 (0)	0 (0)	1
(e) + (f) + (g)	0 (0)	0 (0)	0 (0)	-
(f) + (g)	1 (0.4)	1 (0.5)	0 (0)	1

All of the other data are presented as the number of patients (percentage of all patients).

### Survival

CCI, ADL, and concomitant pneumonia on admission were significantly different in the univariate analysis; therefore, Cox proportional hazards analysis was performed. Age and sex were not included in the study because the participants were ≥65 years, and pneumothorax was more common in males.

In the Cox proportional hazards analysis of ADL (p = 0.09), CCI (p = 0.05), and concomitant pneumonia on admission (p = 0.02), concomitant pneumonia on admission was found to be an independent predictor of in-hospital mortality.

**Fig 1:**
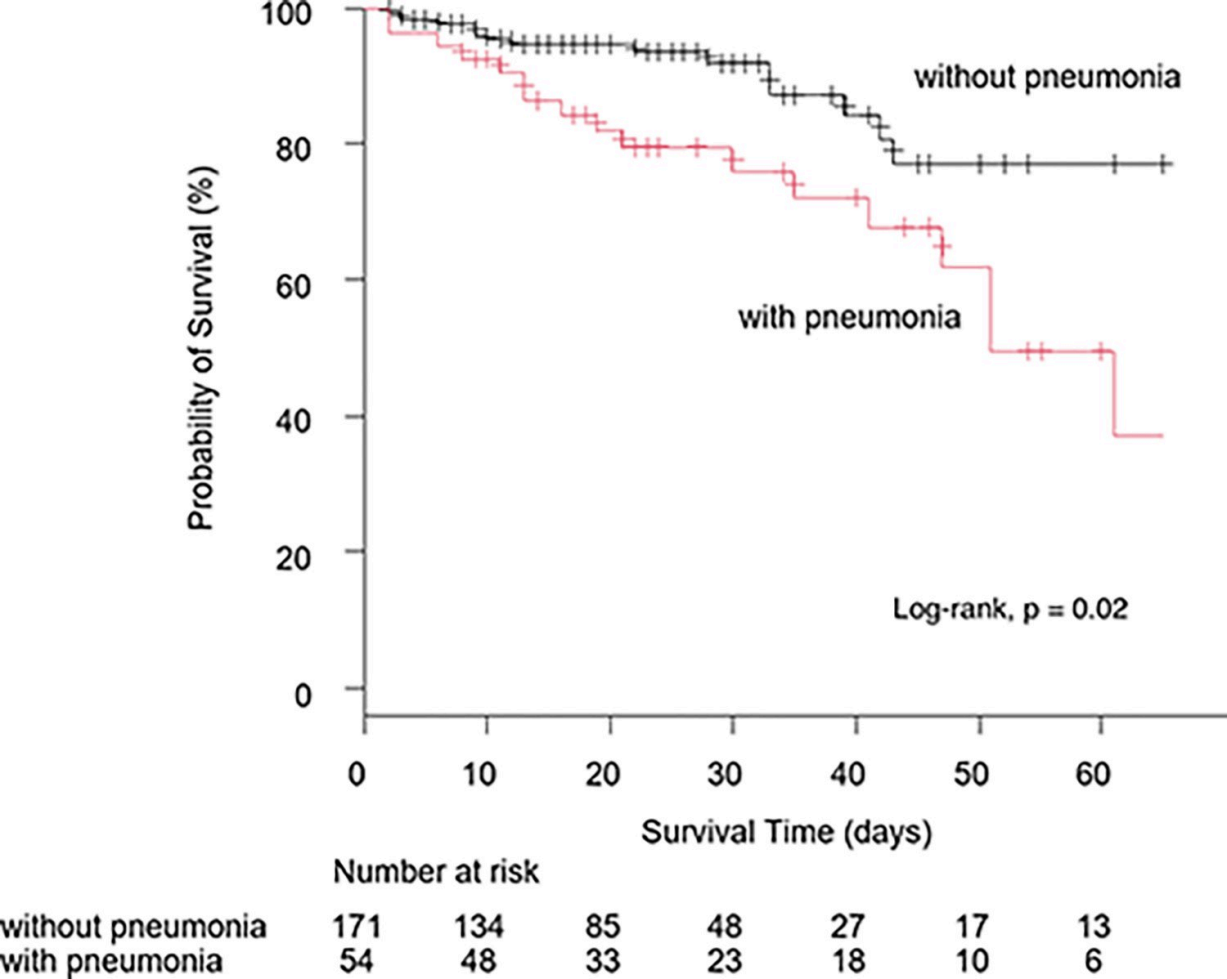
Survival rates of two groups of patients with pneumothorax.

Fig 1 shows the survival rate of the two groups of patients with pneumothorax, one group with concomitant pneumonia and the other without concomitant pneumonia on admission. A log-rank test was performed, and the p-value was 0.02. There was a significant difference in the survival rates between the groups with and without concomitant pneumonia on admission.

Fig 1 shows the survival rates of two groups of patients with pneumothorax: one group had concomitant pneumonia on admission, and the other group had no concomitant pneumonia on admission. A log-rank test was performed, and the p-value was 0.02. There was a significant difference in the survival rates between the groups with and without concomitant pneumonia on admission.

## Discussion

Our study confirmed that older patients with pneumothorax, with or without underlying lung disease, who had concomitant pneumonia on admission should be treated with caution because of the high mortality rate. Although COPD and ILD are considered mortality risks for pneumothorax, this study suggests that concomitant pneumonia on admission may also be a mortality risk factor.

Our study is the second in Japan to investigate pneumothorax in the older population, and it features a larger sample size of 239 older patients, which is notably higher than the sample size of the previous study. Few studies have examined the mortality rate of spontaneous pneumothorax in the older population. The Japan Geriatrics Society and the Japan Gerontological Society have recently published a proposal to define older adults as ≥75 years [9]; however, internationally, older adults are defined as those aged ≥65 years. In this study, the definition of older patients was based on the World Health Organization and United Nations classifications (patients aged ≥65 years) [10]. Since many patients have multiple rather than a single underlying pulmonary disease, we classified the patients more finely for investigation. In this study, 11.7% of patients had no underlying lung disease, indicating that primary pneumothorax can be recognized even in older patients. Emphysema was the most common underlying lung disease. The number of cases was small owing to the detailed classification; therefore, no significant differences were likely to be found. In this study, 22.6% of the patients had concomitant pneumonia on admission. A comparison of the survival and in-hospital death groups showed a significant difference (p < 0.01), suggesting that concomitant pneumonia on admission is a prognostic factor for in-hospital death.

The mortality rate among older spontaneous pneumothorax patients in our hospital was 15%, higher than that in the previous study [4]. One reason is that our hospital has an emergency center with 42 departments that handle tertiary care (where more severe illnesses are concentrated); hence, there are many severe cases with underlying diseases.

Few deaths were directly attributable to pneumothorax. In previous reports, the direct causes of death were COPD, pneumonia, heart disease, empyema, and sepsis [4, 11]. Most of these were due to the progression of comorbidities. Our research suggests that patients with pneumothorax often die of comorbidities rather than pneumothorax itself.

Previous studies have reported that the proportion of patients with both pneumothorax and concomitant pneumonia on admission can be as high as 11% [4, 12–14], whereas, in our hospital, it reached 22.6% (54 cases). Of the patients diagnosed with PSP, 11% (28 cases) had pneumothorax as the sole presenting condition without any underlying pulmonary disease. The mortality rate on admission for patients with concomitant pneumonia was 33% (18/54). Among those with both pneumothorax and pneumonia, only patients without underlying lung disease were included, and the mortality rate was as high as 29% (2/7 cases). Unlike in previous reports, patients with concomitant pneumonia on admission were more frequent with higher mortality rates, suggesting that concomitant pneumonia on admission may be a risk factor for mortality. The development of pneumonia in older patients is most likely due to their poor general health. Pneumonia in older patients is often caused by aspiration pneumonia due to poor swallowing function; however, bacterial pneumonia has also been recognized. Therefore, it is suggested that, even without any other underlying disease, older patients are likely to be in poor general condition and have fewer treatment choices for pneumothorax.

CCI was used to assess the severity of complications. Nineteen diseases were listed in the CCI, and higher scores were assigned to diseases with higher severity. A score was assigned to each disease, and each score was used to evaluate the severity of the complications [15]. When the CCI was used to assess systemic comorbidities, mortality was higher in the group with higher scores. Systemic conditions likely affected the mortality in patients with pneumothorax; however, multivariate analysis showed no significant difference.

Pneumonia is more likely to occur in patients with systemic comorbidities, which may increase mortality rates.

As this study was a retrospective analysis at a single center, it is necessary to confirm the results with a prospective study at multiple centers. The present study included patients aged ≥65 years with underlying diseases, limiting the possible confounding control of age and comorbidities. Further studies are needed to analyze the role of these potential confounders. In addition, pneumothorax is a recurrent disease. It is desirable to keep the patients under observation even after discharge from the hospital and observe them for the period until recurrence and death; however, the patients were observed only during hospitalization. Unlike other countries, Japan does not have a unified medical record system, and without a visit to our hospital, physicians do not know how the patient turned out after discharge. Japan’s medical and insurance systems are unique, and similar studies should be conducted in other countries.

## Conclusions

Although COPD and ILDs are generally considered mortality risks for pneumo-thorax, the results suggest that concomitant pneumonia may also be a mortality risk factor for pneumothorax at admission.

## Supporting information

S1 Checklist STROBE statement—Checklist of items that should be included in reports of observational studies. (DOCX)

S1 Dataset (XLSX)
